# Overview of Epstein–Barr-Virus-Associated Gastric Cancer Correlated with Prognostic Classification and Development of Therapeutic Options

**DOI:** 10.3390/ijms21249400

**Published:** 2020-12-10

**Authors:** Valli De Re, Giulia Brisotto, Ombretta Repetto, Mariangela De Zorzi, Laura Caggiari, Stefania Zanussi, Lara Alessandrini, Vincenzo Canzonieri, Gianmaria Miolo, Fabio Puglisi, Claudio Belluco, Agostino Steffan, Renato Cannizzaro

**Affiliations:** 1 Immunopathology and Cancer Biomarkers, Department of Translational Research, Bioproteomic Facility, Centro di Riferimento Oncologico di Aviano (CRO), IRCCS, 33077 Aviano, Italy; gbrisotto@cro.it (G.B.); orepetto@cro.it (O.R.); mdezorzi@cro.it (M.D.Z.); lcaggiari@cro.it (L.C.); szanussi@cro.it (S.Z.); asteffan@cro.it (A.S.); 2 Pathology, Department of Medicine DIMED, University of Padova, 61-35121 Padova, Italy; lara.alessandrini@aopd.veneto.it; 3 Surgical and Health Sciences, Department of Medical, University of Trieste Medical School, 34100 Trieste, Italy; vcanzonieri@cro.it; 4 Pathology, Department of Translational Research, Centro di Riferimento Oncologico di Aviano (CRO), IRCCS, 33081 Aviano, Italy; 5 Medical Oncology and Cancer Prevention, Department of Medical Oncology, Centro di Riferimento Oncologico di Aviano (CRO), IRCCS, 33081 Aviano, Italy; gmiolo@cro.it (G.M.); fabio.puglisi@cro.it (F.P.); 6 Department of Medicine, University of Udine, 33100 Udine, Italy; 7 Surgical Oncology, Department of Surgery, Centro di Riferimento Oncologico di Aviano (CRO), IRCCS, 33081 Aviano, Italy; cbelluco@cro.it; 8 Gastroenterology, Department of Medical Oncology, Centro di Riferimento Oncologico di Aviano (CRO), IRCCS, 33081 Aviano, Italy; rcannizzaro@cro.it

**Keywords:** Epstein–Barr, gastric cancer, carcinogenesis, targeted drugs

## Abstract

Gastric cancer (GC) is a deadly disease with poor prognosis that is characterized by heterogeneity. New classifications based on histologic features, genotypes, and molecular phenotypes, for example, the Cancer Genome Atlas subtypes and those by the Asian Cancer Research Group, help understand the carcinogenic differences in GC and have led to the identification of an Epstein–Barr virus (EBV)-related GC subtype (EBVaGC), providing new indications for tailored treatment and prognostic factors. This article provides a review of the features of EBVaGC and an update on the latest insights from EBV-related research with a particular focus on the strict interaction between EBV infection and the gastric tumor environment, including the host immune response. This information may help increase our knowledge of EBVaGC pathogenesis and the mechanisms that sustain the immune response of patients since this mechanism has been demonstrated to offer a survival advantage in a proportion of patients with GC.

## 1. Introduction

Epstein–Barr virus (EBV) is associated with several malignancies, including gastric cancer (GC) [1]. EBV is a double-stranded DNA virus that infects and immortalizes B cells. Under some circumstances, it also infects epithelial cells, leading to epithelial cancer, mostly nasopharyngeal cancer (NPC) and GC [2]. 

In vitro, EBV preferentially infects B lymphocytes through the CR2/CD21 receptor, interacting with the EBV envelope glycoprotein gp350 [3], and enters gastric cells through a different receptor [4]. In vivo, the route of entry into epithelial cells is mainly through direct fusion at the cell surface followed by the release of viral capsid into the cell. Ephrin receptor A2 [5] and integrins [6] serve as cofactors for EBV epithelial cell infection. 

Table 1 summarizes the data of receptors and co-receptors used by EBV to gain entry into cells.

B cells and EBV-infected epithelial cells may directly transfer the virus to adjacent epithelial cells across the basolateral membrane, like a cell-to-cell infection mechanism, changing the cellular tropism of EBV from B cells to epithelial cells [10,11,12]. However, Yue et al. demonstrated that in the early stage, the host may resist the EBV cell-to-cell transfer by producing some cytokines and factors of the innate and inflammatory response (i.e., IL-1, IL-6, IL-8, NF-κB, and HSP70) [12]. In particular, IL-1β is a well-known downstream effector of the host antiviral response in the context of the IFN-dependent pathway [13]. To establish infection, EBV has developed a defense mechanism against these host factors. However, all the mechanisms of EBV defense are not completely known and the relatively new cell-to-cell phenomenon remains to be further studied for a better understanding of its clinical implications.

**Figure 1:**
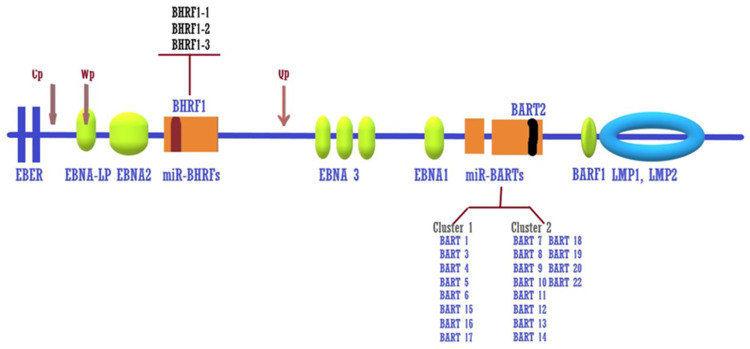
Structure of the EBV genome. Latent proteins (Epstein–Barr virus nuclear antigen (EBNA)1, EBNA2, EBNA3, EBNA-leader protein (LP), BamH1 A reading frame-1 (BARF1), latent membrane protein (LMP) 1 and LMP2 and non-coding RNAs (Epstein–Barr-encoded RNAs (EBERs). miRNAs distributed in three clusters: Bam HI fragment H rightward open reading frame 1 (BHRF1) cluster, BamHI A rightward transcript (BART) cluster 1, and BART cluster 2. Besides, a single miRNA, miR-BART2, is encoded by a region outside the BART cluster. BHRF1 is a homolog to the human Bcl-2 and delays cell death during the EBV lytic cycle replication. miR-BART2-5pencodes antisense to EBV DNA polymerase catalytic subunit BALF5, thus inhibiting the transition of latent to lytic cycle [18]. miR-BART of clusters 1 and 2 are abundantly expressed in latency [19]. EBV EBNA gene transcription during viral latency is regulated by the EBV latency promoters, Cp, Wp, and Qp. Cp and Wp are the origins of the long primary transcript including all the six EBNAs (latency III). In GC cells, Cp and Wp are silenced and only EBNA1 is produced that initiate at the Qp promoter. The latent promoter positions are indicated by arrows.

After gaining entry to the cell, the virus may persist in the cell by establishing a pattern of latency gene expression without the production of virions [14]. In GC, the virus establishes a specific latency. Table 2 lists the known differences in the latency gene expression patterns. The persistence of EBV episome latency in the nucleus of GC cells exclusively requires the production of EBV nuclear antigen 1 (EBNA1) under the control of the Bam H1 Q latency promoter (Qp) (Figure 1). The switch from the EBNA Cp and Wp—hypermethylated during latency—to the Qp promoter leads to the exclusive expression of the EBNA1 antigen. Additionally, about 50% of EBVaGC cases express LMP-2A [15], whereas LMP1 is often absent [11]. miRNA expression largely depends on the latency type and seems to govern the reactivation/latency state of the infected cells. Viral miRNAs were identified quite recently. More recent studies aimed to identify the pattern of miRNAs specifically associated with GC and to decipher their role in GC pathogenesis [16,17].

The main risk factor for GC is *Helicobacter pylori* infection, with a large quote of new cases of noncardiac GC attributed to this bacterium [20]. *H. pylori* preferentially colonizes the antrum and is mainly associated with the histological intestinal type. EBVaGC affects the upper and middle thirds of the stomach and is a poorly differentiated carcinoma [21,22]. The different localizations and histological presentations of EBVaGC and *H. pylori*+ GC suggest that the two GC subtypes may have different pathogeneses, although several studies reported a possible interaction between *H. pylori* and EBV in GC in vitro [23,24,25,26,27,28,29,30]. 

In 2014, a new histologic–molecular classification of GC was proposed, the Cancer Genome Atlas (TCGA) subtypes, which introduced the EBV-associated subtype (EBVa) for GC [31]. The discovery of this new GC subtype led to its prognosis and options for treatment, although today, the clinical benefit of EBVa subtyping is still debated [32,33,34,35,36,37,38].

Reviewing mechanisms linking persistent EBV infection and GC pathogenesis may be helpful for further clarifying the utility of subtyping EBVaGC to improve the prognosis and treatment options. In this review, we present an overview of insights regarding EBV-associated pathogenesis that may contribute to this aim. Particular attention is paid to findings that are propaedeutic for research in innovative or simply more effective therapeutic options.

## 2. Molecular Classification and Prognosis of EBVaGC Subtypes 

Given the histologic and etiologic heterogeneity of GC, no integrated panel of molecular and morphological diagnostic markers has yet been developed for early-stage diagnosis of GC and for selecting the optimal therapeutic approaches. GCs are clinically classified as early, advanced, or metastatic stage to allow for the selection of appropriate treatment options, and based on Laurent (intestinal, diffuse, and mixed types) or World Health Organization (WHO) histological criteria (tubular, papillary, mucinous, and poorly cohesive types) for histological classification. 

Currently, the molecular classifications of GC include the Cancer Genome Atlas (TCGA) subtypes and the Asian Cancer Research Group (ACRG) [31,39]. TCGA categorizes four gastric cancer subtypes: Epstein–Barr-virus-associated (EBVa), microsatellite instable (MSI), chromosomal instable (CIN), and genomically stable (GS) GCs. ACRG classifies GC as microsatellite instability (MSI)-high (MSI-H), microsatellite stable/epithelial-mesenchymal transition (MSS/EMT), microsatellite stable/epithelial/TP53 intact (MSS/TP53+, p53 active), and microsatellite stable/epithelial/TP53 loss (MSS/TP53−, p53 inactive). In addition to these classifications, some molecular markers have a useful impact in GC subtype characterization to lead to more personalized medicine: the presence of HER2 overexpression, which has been associated with a poor prognosis but good response to trastuzumab [40,41]; MSI-H, and EBV-positive tumors as promising candidates for PD-L1/PD-1 immune checkpoint inhibitors [42]; and CDH1 mutations as a marker for the identification and prophylaxis of the hereditary form [43]. The role of other potential biomarkers for prognosis such as Programmed Death-Ligand 1 (PD-L1), circulating DNA (ctDNA) and Tumor Microenvironment Burden (TMB), and promoter hypermethylation of MLH1 gene as a prognostic biomarker for GC with MSI-H [44,45] are further under investigation.

Particular attention merit the MSI-H in resectable GC tumor because, as resulting from the MAGIC study [46] MSI-H was associated with a good prognosis in GC treated with surgery alone but with a negative prognostic effect in patients with resectable GC treated with neoadjuvant. The value of MSI-H as a predictive biomarker in resectable GC was then confirmed in other studies, as reported in a recent meta-analysis [45]. The lower response of MSI-H GC to chemotherapy than other GC subtypes has been attributed to an impaired mismatch repair activity, a biomarker of tumor MSI-H, which hinders cells to recognize DNA-damaged by the cytotoxic drugs and consequently, to induce apoptosis [47]. In addition, MSI-H and cytotoxic treatment may lead to an increased tumor neoantigen production, resulting from a greater rate of mutated gene, and subsequent tumor CD8-positive T-cell infiltration that, however, can be exhausted and inhibited by tumor PD-L1 expression [48]. Consistently, it has been proposed that MSI-H could be more sensible than other molecular GC subtype to immune-check point inhibitors [42]. However, since both DNA-damage and immune response may be dependent of the drug used, thus, further studies are required to better understand the molecular mechanism linking MSI-H with the clinical response to a specific treatment. 

The question of the prognostic impact of EBVaGC was recently investigated in a meta-analysis where the authors concluded that EBVaGC is associated with a lower mortality rate than the other GC subtypes [44]. ACRG classification also supports the good prognosis of EBVaGC, since EBVaGC is more frequently included in the microsatellite stable/TP53+ (MSS/P53+) subtype which, together with the microsatellite-instable (MSI) adenocarcinomas, showed the best survival among the overall GC [39].

Compared to EBV-negative GC, EBVaGC develops more frequently in young men and shows less lymphatic vessel invasion [49,50]. Whether this results in a lower number of metastases remains a subject of debate [1,51]. A recent study focused on young-onset EBVaGC [52]. This study confirmed the associated between EBVaGC and young patients with GC (33%, 40 years old, range 21–45 years) compared to the average-onset of GC (11%, 69 years range 50–90 years). Of note, authors highlighted no difference regarding familiarity with GC, HER2, or MSI-H expression between the two groups of patients but a significant positive association between PD-L1 immunostaining with EBVaGC (50% of cases) and the good clinical prognosis. 

The association between EBVaGc and PD-L1 expression has been shown also in EBVaGC at older onset, but in part because of the low number of EBVaGC cases present in the population; the beneficial response of advanced EBVaGC to PD-1 checkpoint inhibitor after chemotherapy remains to date unclear and must be further investigated [42]. 

**Figure 2:**
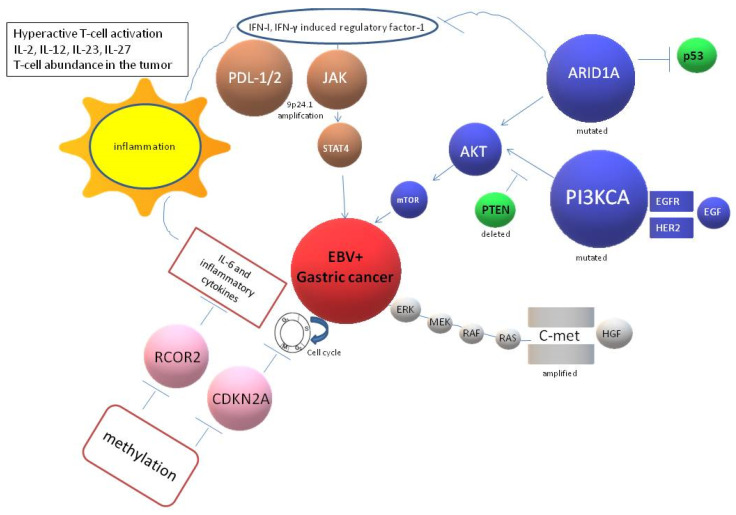
The most important signaling molecules characterizing EBV + GC. There are four main signals known: (1) Gene mutations, mainly affecting the *PIK3CA* and *ARID1A* genes, which lead to an activation of the PI3K/Akt pathway; (2) activation of the tyrosine kinase pathway mainly due to amplification of the *MET* gene; (3) hypermethylation of several genes, in particular, *RCOR2* and *CDKN2A* genes, which leads to the activation of cyclases and an increase in the inflammation surrounding the tumor site with an increase in reactive oxygen species (ROS) production and the release of several cytokines; and (4) amplification of the chromosome 9p24.1, including the *PDCD1L 1-2* and *JAK2* genes.

EBVaGC shows a poorly differentiated histology and a diffuse type [53]. The concordance in molecular data taken from several studies indicates an increased expression of phosphoinositide phosphatidylinositol 3-kinase (PIK3CA), a decreased level of AT-rich interactive domain 1A (*ARID1A*), promoter hypermethylation of the *CDKN2A* coding for the p16 protein, and amplification of 9p24.1 chromosome harboring the Programmed Death ligand genes (*PDCD1L 1-2)*, and Janus-kinase 2 (*JAK2*) [54]. These characteristics together with *SMAD4* and the *APC* mutations are also observed in the MSS/P53+ GC of ACRG subtyping [39]. Figure 2 depicts the most important signaling molecules characterizing EBV + GC. *ARID1A* and *PI3KCA* are the most frequently mutated genes in EBVa GC (5–55% and 80%, respectively) [55]. Amplification mostly occurs for *JAK2* and *PDCD1L1/2* genes (about 11% of cases), and the receptor tyrosine kinase pathway, including *MET* gene amplification, which is also frequent (33% of cases) [31]. 

It is also important to understand the cellular origin of EBVaGC toward increasing our knowledge of the role of EBV in pathogenesis. Several researchers have used diagnostic markers for cell lineage [56,57,58,59,60,61], and CD10 in particular, which is a metalloprotease expressed by enterocytes; mucins produced to protect the tissue from acid and mechanical damages; and tight-junction claudins (CLDN). Overall, the authors indicated that EBVaGC originates from precursors of gastric cells, excluding the intestinal cell type. Since only a fraction of EBVaGC cases is positive for gastric markers and the remaining cases are negative for both gastric and intestinal cell markers, the authors supposed that EBV infection occurs in the precursors of gastric cells and that tumor cells express some mucins only in the late stage during GC growth. Alternatively, and in opposition to Correa’s model for GC, intestinal metaplasia maybe not the precursor lesion of EBVaGC [62]. However, all these studies are in agreement regarding the absence of EBV genome in preneoplastic lesions, which underlines the difference compared with EBV-negative GCs, and mainly with those occurring in the antrum and of intestinal-type. 

## 3. Variations in the EBV Genome Based on Geographical Differences 

The EBV genome has been thoroughly investigated to determine if its variability is due to ethnic or geographic correlates or a specific disease. Traditionally, EBV types were classified based on variations in a few genes such as EBNA2 (the most frequently used), EBNA3, EBNA1, and LMP1 corresponding to two EBV strains: EBV-1 (B95-8 as prototype 1) and EBV-2 (AG876 as prototype 2). EBV-1 was found to be predominant in America, Europe, and Asian countries, but the frequency of the EBV-2 type increases in immunocompromised individuals [63]. EBV-2 predominates in Africa. With advances in genomic and bioinformatic technologies, the entire 175 kb EBV genome has been sequenced, producing evidence of vast EBV variations as the basis of numerous classification studies, e.g., [64,65,66]. Zanella and collaborators, though combined analysis of EBV genetic structural recombination with that of EBV phylogenetic mutations, proposed 12 distinct EBV phylopopulations (EBV-p) based on geographic location and tumor type [64]. Among them, four different EBV-p, including the EBV-p1 group, were found to be associated with different Asian countries, two with Africa, and six were distributed around the world. Of interest, the EBV-p1 group is also associated with gastric cancer [64]. However, their study was limited by the small number of different geographical EBV populations and the tumor types analyzed. Another study that focused specifically on EBV from non-Asian gastric cancer highlighted some shared mutations among gastric cancer samples independent of geographical origin. These shared mutations mapped to antigen epitopes, which suggests EBV immune evasion may be involved in the development of EBVaGC [65]. In recent years, a higher prevalence of EBVaGC was evidenced in Latin America compared to Europe and Asia [66,67]. The higher prevalence was related to the Hispanic–European migration [66]. In the population of Chile and Peru, Corvolan et al. [66] highlighted the segregation of gastric cancer with the European-Asian BamHI restriction site I region of the EBV genome, which includes the BARF1 and the miR-BARTs transcripts. Therefore, they proposed a common ancestry of EBV between Latins and Europeans followed by a disrupted-co-evolution (XhoI loss site in the LMP1 gene) in Latins [66]. Although this hypothesis is intriguing, further studies are necessary to demonstrate the role of a specific LMP1 region with gastric cancer risk. 

Taken together, the data suggest the role of specific EBV variants which are associated with an increased risk of GC. Additional studies should be performed to determine the biological significance of the EBV sequence variations for clinical applications and to study the potential attenuation of antiviral T-cell immunity caused by these viral strain variations. 

## 4. EBV Latency and Epigenetic Activity

The latent gene profile in EBVaGC is specific, showing substitution of the oncogenic role of LMP1 and EBNA2 proteins by that of the BARF1 protein [68] and, in some cases, of LMP2A [69]. BARF1 plays an oncogenic role by reducing the expression of tumor-suppressor genes in host cells [68,70]. Besides, secreted hexameric BARF1 was able to induce an immunosuppressive milieu through action on macrophages since it binds to human macrophage colony-stimulating factor (M-CSF) [71]. LMP2A has been reported to induce stemness phenotype, resulting in the alteration of motility, inhibition of differentiation, and anchorage-independent cell growth, [72,73,74]. However, conversely, to EBNA1, EBERs, and mi-BARTS, which are highly expressed in EBVaGCs, LMP2A expression were reported only occasionally in EBVaGC [75].

EBERs, which are EBV-encoded small RNAs, were shown to confer resistance to apoptosis by inducing the expression and secretion of EBV insulin-like growth factor [76]. More recently, EBV was found to also express miRNAs. The BHRF1 region encodes 4 miRNAs, and the BART region encodes the remaining 40 miRNAs; miRNA--BHRF1 is expressed only in the lytic phase, whereas the miRNA-BARTs are characteristically expressed at high levels in the latent form in EBVaGC and were found to influence mainly apoptosis and the immune response [77]. A recent study identified a mutation in the promoter of BART that could increase the production of miRNAs in EBVa NPC but the same single polymorphism (SNP) was scarcely found in EBVaGC [78]. Thus, today the reason for the high level of miR-BARTs in EBVaGC remains unclear. 

Numerous studies revealed that various genes involved in GC are targets of EBV miRNAs and are pro-tumorigenic, some of which are detailed in Table 3. Particular attention is paid to miR-BART5-3p, and miR-BART1-5p in EBVaGC [79], since they were found to bind and facilitate degradation of the important tumor suppressor P53 mRNA [80]. Besides, miR-BART1-5 and miR-BART9 are involved in cell growth and migration by inducing the reduction of important proteins like E-cadherin, which are necessary to maintain epithelial cell cohesion [81,82].

Conversely, few EBV miRNAs have demonstrated oncosuppressor potential. For example, BART15-3p was found to promote apoptosis and immune responses [17], and BART6-3p was found to reverse the epithelial-mesenchymal phenotype and inhibit tumor invasion and migration [83]. A study whereby the AGS gastric cell line, infected with EBV or without infection, was inoculated into immunodeficient mice, resulting in significant fold-change of protein and RNA representing at least 240 genes in the tumors [84]. Some of the upregulated genes acted to increase DNA repair and RNA transcription regulators, but most others were proto-oncogenes or kinases. The downregulated genes were mainly involved in the movement, survival, and proliferation of tumor cells and the host immune response. In vivo, the tumor infected with EBV showed an increase in hematopoietic progenitor cells and hemorrhaging in mice, mediated by AKT and the hypoxia-inducible factor-1-alfa (HIF1-α) pathways [84]. Additionally, the study demonstrated the specific involvement of histone deacetylase 1 (HDCA1) as an important epigenetic mechanism of gene regulation in EBVaGC.

Hypermethylation is another hallmark of EBVaGC, but this mechanism does not involve the *MHLH1* gene—a component of the mismatch repair system strongly associated with the GC MSI-H subtype [85]. Of note, among the genes silenced by methylation, the *REST* corepressor (Rcor)-2 (*RCOR2*) gene was found to be silenced in 100% of EBVaGC and 0% of EBV-negative GC. *RCOR2*, like *ADRIA*, is a regulator of chromatin and suppresses the expression of several genes; it plays a key role in the protective inflammatory program and the longevity of cells, is highly expressed in embryonic stem cells (ESCs). The methylation of the *CDKN2A* promoter region is also frequent, resulting in the downregulation of CDKN2A (p16) cyclin-dependent kinase inhibitor [86]. Several studies have focused on identifying methylated genes in plasma or serum samples that could be used as novel non-invasive diagnostic, prognostic, or survival markers in GC [87,88,89,90].

## 5. Exosomes and Autophagy in EBVaGC

Recent research has focused on the impact of exosomes and autophagy, which have emerged as a novel and potentially important mechanisms for the initiation, progression, and invasion of cancer. Regarding EBVaGC, the mechanisms of exosome entry, specific cell targeting, and cargo delivery are still under investigation. Exosomes may transport EBV EBER RNAs [76,104] and the incorporation of the EBV glycoprotein gp350 allows exosome cargo delivery in B cells through the CD21 receptor [105]. Researchers are now focused on investigating the potential and challenges of exosome products as diagnostic markers and therapeutic targets for EBVaGC. 

**Figure 3:**
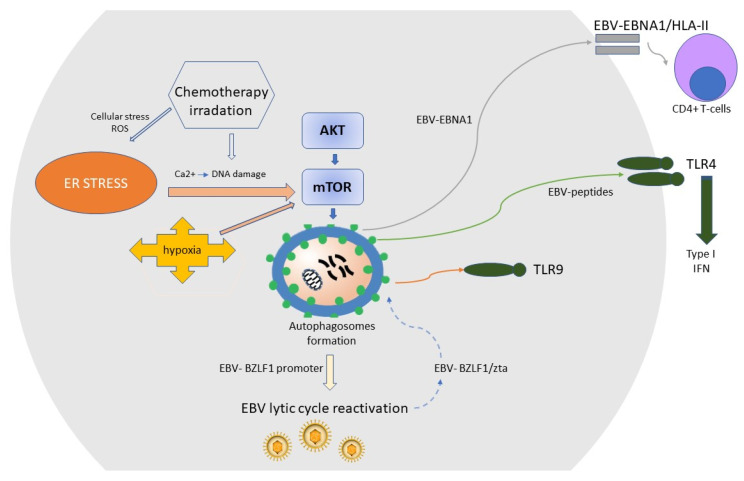
The cellular degradation pathway of autophagy plays a fundamental role in immunity. Environmental perturbations (endoplasmic reticulum (ER) stress, hypoxia) as well as chemotherapy and radiation, by inducing DNA damage and cellular stress, inhibit AKT/mammalian target of rapamycin (mTOR) and activate autophagy. The autophagy machinery interfaces with most immune signaling molecules involved in the development, homeostasis, and survival of inflammatory cells, including macrophages, neutrophils, T lymphocytes, and B lymphocytes. Disturbance of ER homeostasis that cannot be rescued by the unfolded protein response (UPR) results in autophagy, which is largely attributed to the inactivation of the mTOR and downregulation of AKT/mTOR pathway, which is an important signaling pathway in the development of GC. Autophagy is necessary for recognition of the EBV-EBNA1 antigen by CD4+ T cells via HLA-II and EBV-mediated activation of the Toll-like receptor (TLR) and plays a role in the reactivation of the EBV lytic cycle. The EBV-BZLF1 protein, also known as Zta, creates a positive loop with autophagosomes to maintain the EBV lytic cycle. IFN, type I interferon.

Regarding autophagy, various EBV antigens may be degraded by xenophagy (autophagy of microbes), and the resulting peptides are used to form the antigen/HLA complexes that are presented to immune cells in a functioning immune system. The best-known process is the recognition and presentation of EBV EBNA 1 antigen by HLA class II to CD4+ T cells (Figure 3) [106]. Autophagy also activates Toll-like receptor (TLR) signaling through the delivery of EBV peptides as ligands for TLRs, resulting in the production of type I IFN [107] or the activation of specific TLRs [108]. Moreover, some EBV proteins, like the EBV-Zta of the early lytic phase, can manipulate the host autophagic machinery for its self-advantage [109]. Figure 3 displays some of the interactions between autophagosomes and EBV. Of note, since autophagy is involved in the host immune response against GC, presenting EBV antigens and probably involved in reactivation of the EBV lytic cycle, pharmacologic modulation of autophagy has been proposed as a new type of therapy for EBVaGC [110].

## 6. EBV Lytic Reactivation 

EBV’s role in gastric carcinogenesis is poorly understood and continues to be investigated. Recent studies have drawn attention to the potential reactivation of the EBV lytic cycle in addition to the EBV latency expression pattern. Researchers found that local inflammation of the stomach is associated with EBV lytic cell reactivation, probably from the B-cell reservoir, with the cell-to-cell model for EBV transfer in different cell types consequently leading to a higher EBV infection rate for epithelial cells. Since lytic BZLF1 gene expression was found in some EBVaGC cells, researchers supposed that lytic infection also occurs in some GC cells, further increasing EBV propagation [111]. Hypoxia, a condition associated with GC, and radiation therapy were also found to be able to reactivate EBV infection from its latent state [78,112]. EBV infection is found more often in carcinomas of the stomach after surgery than in the intact stomach or the pre-neoplastic conditions [113]. Overall, the data suggest that the EBV lytic state and propagation may occur under particular conditions of stress and that the resulting inflammation and tissue damage might favor GC progression. The data also indicate that EBV may infect the well-differentiated epithelial cells of the stomach. However, whether the reactivation of the EBV lytic cycle plays a role in EBVaGC carcinogenesis, prognosis, or response to therapy remains to be investigated. 

## 7. EBV Interference with Host Immune Responses 

High MHC-II expression in EBVaGCs suggests that this tumor shows a more important role in antigen presentation than EBV-negative GC [65]. Nonetheless, although, EBVaGC recruits numerous reactive immune cells [114], specifically CD8+ T cells and macrophages [115], numerous strategies are used by EBV to evade the immune response. Among the latest discoveries are the production of EBV miRNAs, the use of host-secreted exosomes to transfer immunosuppressive activities to neighboring cells, and the alteration of autophagy and cell metabolism. 

Hooykaas et al. showed that EBV miRNA BART16 can inhibit the generation of the type I interferon gene response, which is one of the most important antiviral adaptive immune responses produced by humans and, thus, increase in replication of the virus [116]. Nachmani et al. showed that miRNA BART2-5p may target the stress-induced immune ligand MHC-I polypeptide-related sequence B (*MICB*), thus inhibiting the ligand/receptor interaction that activates natural killer (NK) cell recognition of damaged cells [117]. Ross et al. demonstrated that BART11-5p, produced on EBV-infected B cells, can block the development of memory B cells in the germinal center, thus delaying the B-cell immune responses [118]. Alteration in the B-cell immune response might influence the adaptive immune response against infected cells including EBVaGC, though this is today a hypothesis that remains to be demonstrated. Haneklaus et al. showed that miRNA BART15 may prevent the accumulation of the inflammasome NLRP3 complex, which is usually activated in response to intracellular or extracellular signals of stress and consequently induces inflammation through the release of cytokines such as IL-1β and IL-18 [119].

Additional important targets of miRNAs include several host genes and EBV genes reported to maintain EBV in a latent phase and inhibit autophagy (e.g., by reducing the transport of EBV antigens to lysosomes or by inhibiting peptide degradation into endosomes and lysosomes), resulting in further reductions in the processing and presentation of viral antigens [120]. 

Further BART miRNAs were found to block natural killer (NK) and T-helper 1 (Th1) cell recruitment and inhibit lymphocyte differentiation and activation via interference with different immune checkpoints (e.g., CXCL11/CXCR3 [121] and IL-1/IL1-R [122]) or through the production of IL-12, an important cytokine involved in the regulation of adaptive and innate immunity [123]. 

Furthermore, EBVaGC cells in concomitance to latent-related gene expression, frequently express the BZLF1 gene involved in the lytic cycle [121], which was associated with the downregulation of MHC-II-related antigen presentation [124,125]. 

Another important mechanism by which EBV reduces host immunity is to alter the metabolism of infected cells, thus modifying the T-cell immune response. When T cells encounter antigens on antigen-presenting cells, the T cells initiate glycolysis, which requires the presence of oxygen to obtain more energy for T-cell division and effector functions. However, during chronic antigen stimulation, as in cancer, tumor cells increase the uptake of glucose for aerobic glycolysis (Warburg effect) and increase the mitochondrial activity and lipid metabolism, thus decreasing glucose and lipid availability in the microenvironment for T cells. Moreover, tumor cells are known to produce higher amounts of ROS than normal cells, and the release of ROS into the microenvironment increases the proportion of cells more resistant to ROS products. This leads to an accumulation of the tumor-infiltrating Treg cells to the disadvantage of the cytotoxic CD4+ T cells [126]. Consequently, the T-cell effector response was found to be less vigorous and more exhausted in the tumor microenvironment [127]. However, the alteration of metabolism in EBVaGC remains to be determined. A comprehensive omics study by Sang et al. offered an important contribution to filling this knowledge gap in the future [128].

## 8. Targeted Therapies for EBV + GC

EBV positivity is now well accepted as a consolidated marker for the diagnostic classification of GC.

To date, EBER in situ hybridization on biopsy samples remains the gold standard to determine the EBV-positive GC subtype [129]; the quantification of EBV viral load by quantitative DNA amplification of blood samples and tumor tissue have been proposed in the last years as promising tests for the early diagnosis, evaluation of the latency/reactivation status of the virus, and prediction of recurrence and chemotherapeutic response to GC treatment [130]. 

Because of its low frequency (commonly less than 5–10% of gastric cancer), the clinical utility of EBV as a prognostic marker and a target for therapeutic options in gastric cancer has been the object of several studies with conflicting results. Some studies reported no differences in survival between EBV-positive and EBV-negative GC after surgery and/or conventional chemotherapy, whereas other studies supported the association between EBV infection and favorable prognosis [32,33]. Notably, patients with advanced EBVaGC responded better to chemotherapy with 5-fluorouracil and platinum [34,35,36].

Although it has yet to be demonstrated, the immune response from the presentation of EBV-related antigens has been hypothesized to help improve prognosis in addition to the effects of chemotherapy-induced neoantigens [31,37,38]. Conversely, decreased efficacy of the immune response has been reported in elderly patients [131], in patients with long-lasting responses [132], and those with more advanced disease [133]. 

To further improve the results obtained with conventional chemotherapy, several therapeutic approaches aimed at specific targets (i.e., immune response, demethylation, EBV replication, miRNAs, antisense oligonucleotide, and receptor tyrosine kinase) are being studied in patients with EBVaGC. Here, we report those that are the most promising. 

High CD8+ T-cell infiltration in the tumor microenvironment is usually associated with a better prognosis, but this favorable factor is often countered by the presence of high levels of T-cell inhibitor signals. The most frequent inhibitory signal found in EBV-positive gastric cancer is high PD-L1 expression resulting from 9p24.1 amplification (about 11% of cases) [134], which leads to immune resistance and reduced survival of patients [135]. EBV gene expression may directly regulate PD-L1 and PD-L2 gene expression [136,137]. The mechanisms by which EBV reduces PD-L1 expression remain to be elucidated in EBVaGC, since EBV LMP-1 and EBNA-2 genes, which can increase PD-L1 expression, are not expressed in EBVaGC. It is supposed that T cells in the tumor microenvironment may contribute to the high expression of PD-L1 in EBVaGC by releasing IFN-γ in response to EBV-infected cells [138]. 

Because of the high PD-L1 expression found in GC, PD-1/PDL-1 blockade has been proposed as a target for therapy. Metastatic EBV + GC patients who were treated with pembrolizumab, a PD-1/PD-L1 inhibitor, showed good responses, and the favorable prognosis is reliant on high levels of PD-L1 and the number of infiltrating CD8+ T cells in the tumor [139]. However, since not all EBVaGCs have high PD-L1 expression, positivity for both EBV and PD-L1 must be tested before treating patients. Nonetheless, and though this study is in phase II, 100% of EBVaGC cases were responsive to PD-L1 checkpoint inhibitors, whereas 85% of MSI-H EBV-negative cases were responsive. However, these findings need further investigation in a larger cohort of EBV-positive cases. In this regard some studies have been performed in the last years, and are still ongoing. For instance, Kubota et al. found improvement in progression-free survival (PFS) time in 33% of advanced EBVa GC subtype following anti-PD-1 therapy compared to earlier-line chemotherapy [42], whereas, Kawazoe et al., by using a combination of pembrolizumab plus lenvatinib, an inhibitor of VEGF receptor that could improve the anti-PD-1 activity, found an objective response in the unique case of EBVaGC tested [140]. 

Tyrosine kinase receptors are involved in several tumor growth and metastatic pathways as some of them are upregulated in GC. Targeting therapies against these molecules in GC have been intensively investigated. To date, better success in treating GC has been obtained using the monoclonal antibody trastuzumab and the antibody-drug conjugate trastuzumab deruxtecan (DS-8201) against human epidermal growth factor receptor 2 (HER2 receptor) [141,142], and the monoclonal antibody ramucirumab against vascular endothelial growth factor-2 (VEGFR-2) [143]. 

Another important tyrosine kinase inhibitor strongly associated with EBVaGC is PI3K, which binds several ligands (including HER2 and EGFR) [144]. Mutations in exons 9 and 20 in *PI3K* catalytic subunit alpha (*PIK3CA*) as well as mutations in phosphatase and tensin homolog (*PTEN*), *AKT1*, *AKT2*, and *AKT3* lead to deregulation of the PI3K–Akt–mTOR pathway; they have been proposed as biomarkers to test novel compounds and dosing schedules [145]. They are also associated with peritoneal recurrences [145,146,147], resulting in AKT and PTEN having immunohistochemistry prognostic value for patient survival [148]. Circular RNA AKT3 (circ AKT3) was found to be associated with PIK3/Akt activation in GC and resistance to cisplatin [149,150,151]. Cisplatin damages cancer cell DNA; thus, EBV, through modulating the activation of the PI3K/AKT pathway, enhances DNA repair mechanisms that may counteract the therapeutic effect of cisplatin. If this observation can be confirmed in vivo, circAKT3 could be a promising prognostic marker to evaluate cisplatin resistance in patients with advanced GC. Some PI3K/Akt/mTOR inhibitors (e.g., idelalisib (CAL-101) and copanlisib) have been Food and Drug Administration (FDA)-approved and are in clinical testing [152]. Some clinical trials of PI3K inhibitors (BKM120, BYL719, and GSK2636771) and Akt inhibitors (MK2206, GSK2110183, and GDC-0068; Table 3) have been performed in patients with advanced and metastatic stage GC. Unfortunately, despite good results in preclinical studies, no significant results have yet been reported.

The tumor suppressor *ARID1A* gene, a component of the yeast switch/sucrose non-fermenting (SWI/SNF) complex, acts as a chromatin remodeling factor affecting gene promoters and thus modulating the transcription, replication, recombination, and repair of the underlying DNA. Loss of or low expression of *ARID1A* has been associated with lymph node metastasis and poor prognosis in GC [153]. Moreover, an association was reported between a high frequency of *ARIDIA* mutations and overexpression of the PD-L1/L2 [154] and modulation of *TP53*, which is rarely mutated in EBVaGC compared with the other GC subtypes [155]. *ARID1A* and EBV infection were found to be related to a reduction in type I interferon (i.e., IFN-α/β) production [156] and with an increase in PD-L1 expression [157]. Because of the important functions of these molecules in EBVaGC, a strategy that combines PD-L1 and *ARID1A* targets is considered to have the therapeutic potential [154].

Although several antibodies have been developed in the last decade against antigens expressed on GC cells (e.g., cetuximab and trastuzumab) or in the tumor microenvironment (e.g., nivolumab, pembrolizumab, and ramucirumab) to treat patients with refractory or recurrent GC, the obtained clinical benefits are still modest [158,159] and research to obtain more effective molecules are still ongoing. Promising examples include zolbetuximab and claudiximab, which are newly developed monoclonal antibodies directed against the tight junction protein claudin 18.2 (CLDN18.2), which is strictly confined on the surface membrane of differentiated epithelial cells of the gastric mucosa [160].

Another feature displayed by EBVaGC is the increased hypermethylation level of multiple genes in tumor cells. For this, several compounds (e.g., decitabine, flavonoids, and zebularine) are being explored in preclinical and clinical trials as treatments to reverse the hypermethylation of genes induced by EBV infection [161,162].

Lytic induction therapy is a rational means to promote the destruction of infected cells and to increase immune recognition. Radiation therapy and selected drugs inducing lytic viral gene expression and/or autophagy (e.g., ganciclovir, histone deacetylase, and butyrate) were found to be effective for treating some EBV-infected cancers such as nasopharyngeal carcinoma and Hodgkin’s lymphoma. Thus, they are also promising for EBVaGC and under investigation. 

Several studies investigated the association between miRNAs and sensitivity/resistance to chemotherapeutic drugs to predict response to target therapies of tumors including GC [163]. Based on these results, silencing of miRNA (via siRNA) has been proposed as a potential new therapeutic approach in human diseases, but most studies are still at the preclinical stages [164]. The advantages of using miRNA instead of conventional drugs include their pleiotropic and concomitant function on multiple genes in the same cell, the capability for delivery at high levels into tumor cells, the use of the same drug for different types of cancer, and the time- and cost-effectiveness of the methods for production [165]. Today, the approach is still in its infancy, with no ongoing trials for EBVaGC therapy. A similar approach involves using antisense oligonucleotides which are complementary to messenger RNA (mRNA), instead of miRNA for intracellular genes overexpressed in cancers, to mediate change in the behavior of malignancies [166]. To date, FDA has approved antisense drugs for some non-tumor genetic disorders (e.g., [167,168,169]). Using antisense oligonucleotides against angiogenetic factors is one of the promising methods for therapeutic intervention with an antisense approach [170]. KDR/Flk-1-ASO, an antisense oligonucleotide against the vascular VEGFR, showed efficacy in a model of the human GC cell line in nude mice [171]. To the best of our knowledge, there are no antisense oligonucleotide clinical trial(s) underway for EBVaGC. 

Patients with GC who carry heterogeneous and rare mutations that can benefit from targeted therapies may also benefit from treatment choice guided by liquid biopsy, which is a promising noninvasive and repeatable approach used to obtain tumor genotyping information. Liquid biopsy refers to the analysis of tumor-derived materials shed in the bodily fluid of cancer patients, such as circulating tumor cells and circulating tumor DNA (ctDNA). In GC, heterogeneity has been widely documented as one of the major hurdles for the success of targeted therapy. Since ctDNA is released from different multiple tumor sites, including metastasis, it can reduce the problem of intratumoral heterogeneity [172]. Accordingly, the matched profiles of primary tumors, metastasis, and ctDNA from GC patients enrolled in the personalized Antibodies for Gastro-Esophageal Adenocarcinoma (PANGEA) clinical trial confirmed the high agreement between metastatic and ctDNA profiles in 87.5% of gene alterations [173,174]. Other studies reported the clinical utility of detecting *HER2* in ctDNA to predict the response to trastuzumab treatment and to decipher the mechanisms of resistance [175,176,177]. 

Concerning EBVaGC, the related targeted next-generation sequencing tumor profiling of ctDNA identified the enrichment of *PIK3CA* mutations in these tumors. These results were in line with the heterogeneity of *PIK3CA* mutations found both in the primary tumor and in the matched lymph node and/or metastatic biopsies from the same patients, in agreement with the TCGA classification of EBVaGC [178,179]. EBV DNA has also been tested as a marker in ctDNA analysis to evaluate the tumor burden and monitor specific treatment response in EBVaGC [180,181].

Overall, the liquid biopsy findings show promise for detecting predictive and prognostic biomarkers in EBVaGC, but this approach is still in its infancy and requires further investigation.

## 9. Conclusions

A significant association between EBV infection and GC has only recently been identified, and the mechanisms of EBV-induced carcinogenesis are therefore poorly understood. Novel insights into EBVaGC subtype characteristics include the amplification of chromosome 9p24.1 (JAK/PD-L1), methylation, and regulation of cellular autophagy, EBV lytic cycle reactivation, EBV-miRNAs, exosomes, and immunosuppression. Overall, these aspects must be better understood to further our knowledge of the pathogenesis of EBVaGC and to help develop promising novel therapeutic approaches.

## Figures and Tables

**Table 1:** Epstein–Barr virus (EBV) protein and receptor interactions.

EBV Protein	Function	Cellular Receptor(s)	Reference
BMRF2	A lytic protein of EBV; attachment to epithelial cells at the basolateral surface	β1 or α5β1 integrins	[6]
gp350/220	Attachment to cells	CR2/CD21 the major receptor.	[3]
gp42 *	Binds B cells, the entry-receptor-binding protein in epithelial cell block gH/gL interaction with integrins	MHC class II	[7]
gH/gL- gB	The heterodimer is necessary for membrane fusion. Regulates fusion with epithelial and B cells	αvβ6 and αvβ8 integrins	[8]
gH/gL	Receptor for entry into B cells and epithelial cells	EphA2, an erythropoietin-producing hepatocellular (Eph) family member of receptor tyrosine kinases	[5]

* gp42 inhibits entry into epithelial cells but virus produced in B cells is deficient in gp42, so is thus able to infect epithelial cells, resulting in the transfer of infection mediated by B lymphocytes. Gp42 probably interacts with MHC-II in B cells resulting in changes in conformation in the viral attachment protein. When virions are produced by epithelial cells, gp42 is highly expressed and allows infection of B cells [9]. Gp, glycoprotein, gH/gL-gB, EBV glycoprotein.

**Table 2:** Expression patterns distinguishing the different forms of EBV latency.

Cell Type	Latency	BHRF1/miR	BART/miR	BARF1	EBNA						LMP			EBER
					1	2	3A	3B	3C	LP	1	2A	2B	
B cell	I	+	+	−	+	−	−	−	−	−	−	−	−	+
	II	+	+	−	+	−	−	−	−	+	+	+	+	+
	III	+	+	−	+	+	+	+	+	+	+	+	+	+
GC cell	I (II)	−	+	+	+	−	−	−	−	−	−	+/−	−	+

EBNA, Epstein-Barr nuclear antigen proteins; LMP, latent membrane proteins; BART, BamH1 A rightwards transcripts; BARF1, BamHI A rightward frame 1; BHRF1, BamHI fragment H rightward open reading frame 1; EBER, Epstein-Barr virus encoded; GC, gastric cancer; Presence (+) absence (−).

**Table 3:** Functional roles of some EBV genes and RNAs in GC.

EBV Latent Genes	Mechanism of Tumorigenesis	References
BARF1	Induces anti-apoptotic *Bcl-2* and *cyclin D* genes Damages macrophage colony-stimulating factor	[68,71]
EBNA 1	Induces ROS accumulation and impairs response to DNA damage Suppression actives EBV reactivation	[91,92]
LMP2A	Activates *NF-κb*, *Notch*, *and PI3K/Akt* pathways Downregulates HLA Upregulates miR-155-5p	[93,94,95]
**EBV RNAs**		
EBER	Induces IGF-1, an insulin-like growth factor	[76]
**miR-BARTs**		
BART1-5p	Targets glucosaminyl(N-acetyl) transferase 3 (*GCNT3*) pathway	[96]
BART9	Decreases E-cadherin expression	[82]
BART5-3p	Increases the degradation of P53 and inhibits the tumor suppressor DICE1 gene	[80,82,97]
BART5	Targets PUMA	[98]
BART9, -11, -12	Downregulates Bim expression	[99]
BART4-5p	Suppresses Bid	[100]
BART20-5p	Interacts with the 3′ UTR region of the *Bcl-2*-associated agonist of cell death (*BAD*) gene	[101]
BART16	Suppress type I interferon (IFN) signaling	[102,103]
BART15-3p BART6-3p	Oncosuppressor action	[17,83]

## Abbreviations

EBV	Epstein-Barr virus
GC	Gastric cancer
EBVaGC	Epstein-Barr-positive gastric cancer
*H. pylori*	Helicobacter pylori
EBNA	Nuclear antigen proteins
LMP	latent membrane proteins
BART	miR-BamH1 A rightwards transcripts
EBERs	Epstein–Barr-encoded RNAs
MHC	Major histocompatibility complex
NPC	nasopharyngeal cancer
IL	Interleukine
IFN	Interferon
TCGA	Cancer Genome Atlas
ACRG	Asian Cancer Research Group
MSI	Microsatellite unstable
MSS	Microsatellite stable
EMT	Epithelial-mesenchymal transition
PD-(L1)	Programmed Death-(Ligand 1)
